# Acute kidney injury: exploring endoplasmic reticulum stress-mediated cell death

**DOI:** 10.3389/fphar.2024.1308733

**Published:** 2024-02-12

**Authors:** Cong Cheng, Yuan Yuan, Fang Yuan, Xin Li

**Affiliations:** ^1^ Department of Pharmacy, The Third Hospital of Changsha, Changsha, Hunan, China; ^2^ Hunan Provincial Key Laboratory of Anti-Resistance Microbial Drugs, Changsha, Hunan, China; ^3^ Xiangya Hospital, Central South University, Changsha, Hunan, China; ^4^ Department of Emergency, Changsha Hospital of Traditional Chinese Medicine (Changsha Eighth Hospital), Changsha, Hunan, China

**Keywords:** endoplasmic reticulum stress, acute kidney injury, autophagy, ferroptosis, apoptosis, pyroptosis

## Abstract

Acute kidney injury (AKI) is a global health problem, given its substantial morbidity and mortality rates. A better understanding of the mechanisms and factors contributing to AKI has the potential to guide interventions aimed at mitigating the risk of AKI and its subsequent unfavorable outcomes. Endoplasmic reticulum stress (ERS) is an intrinsic protective mechanism against external stressors. ERS occurs when the endoplasmic reticulum (ER) cannot deal with accumulated misfolded proteins completely. Excess ERS can eventually cause pathological reactions, triggering various programmed cell death (autophagy, ferroptosis, apoptosis, pyroptosis). This article provides an overview of the latest research progress in deciphering the interaction between ERS and different programmed cell death. Additionally, the report consolidates insights into the roles of ERS in AKI and highlights the potential avenues for targeting ERS as a treatment direction toward for AKI.

## 1 Introduction

Acute kidney injury (AKI) is a common but usually under-recognized disorder with a high global morbidity and mortality rates and consequent societal burden (Kellum et al., 2013). According literature, the morbidity of AKI has been estimated to be 3.0%–18.3% in high-income countries, and 21% in low to middle income countries (Hoste et al., 2018). In ICU patients, septic AKI account for about half of the total patients (Uchino et al., 2005). There is a significant difference in terms of mortality rates between patients with and without AKI, the mortality rate associated with AKI (20.9%–56.8%) is higher than non-AKI patients (8.4%) (Ostermann and Chang, 2007). In STEMI patients with AKI, the nosocomial mortality is 10 times higher compared to patients without AKI (Cosentino et al., 2021). In addition, AKI is also becoming an important complication in hospitalized children (Chanchlani et al., 2019). Typical histopathological characteristics of AKI include acute renal tubular damage, less commonly tubulointerstitial injury, and vascular dysfunction (Lombardi et al., 2016). Clinically, ischemia-reperfusion injury (IRI), sepsis and nephrotoxic agents are main reason in the development of AKI. Since AKI links with poor prognosis and high mortality in patients, effective treatment for AKI advances in therapies for AKI have been limited, it is an urgent need to clarify the mechanism of AKI and identify therapeutic interventions. Several research studies have indicated that the endoplasmic reticulum stress (ERS) plays a crucial part in the pathophysiology of acute kidney injury (AKI) caused by different factors. AKI research has increasingly prioritized the ERS response (Linkermann et al., 2014; Zhu et al., 2017; Liu et al., 2019).

The endoplasmic reticulum (ER) is an indispensable organelle found in eukaryotic cells (Zhao et al., 2013). Typically, ER serves as the essential place for protein folding. Protein folding quality control is monitored and maintained by the unfolded protein response (UPR) (Lee, 2005; Hetz, 2012). When the body is subjected to external adverse factors, improper protein folding and processing lead to a large stack of unfolded proteins in the ER lumen, causing a stress response named ERS (Kaufman, 1999; Senft and Ronai, 2015). Simultaneously, UPR is activated as a homeostatic network to counteract the adverse external condition and facilitate protein folding, alleviate ERS, and return ER to homeostasis (Frakes and Dillin, 2017; Hung et al., 2019). Nevertheless, in cases of severe and prolonged ERS, UPR is unable to restore equilibrium in the ER, leading to the occurrence of cellular demise.

Thus, in the review, we analyzed the mechanism pathways of ERS and the different deaths caused by ERS, investigated the crucial role of ERS in the development of AKI (IRI, sepsis and nephrotoxic agents), explored the treatment strategy for AKI is by targeting ERS.

## 2 ERS: three pathways of UPR in mammals

**FIGURE 1:**
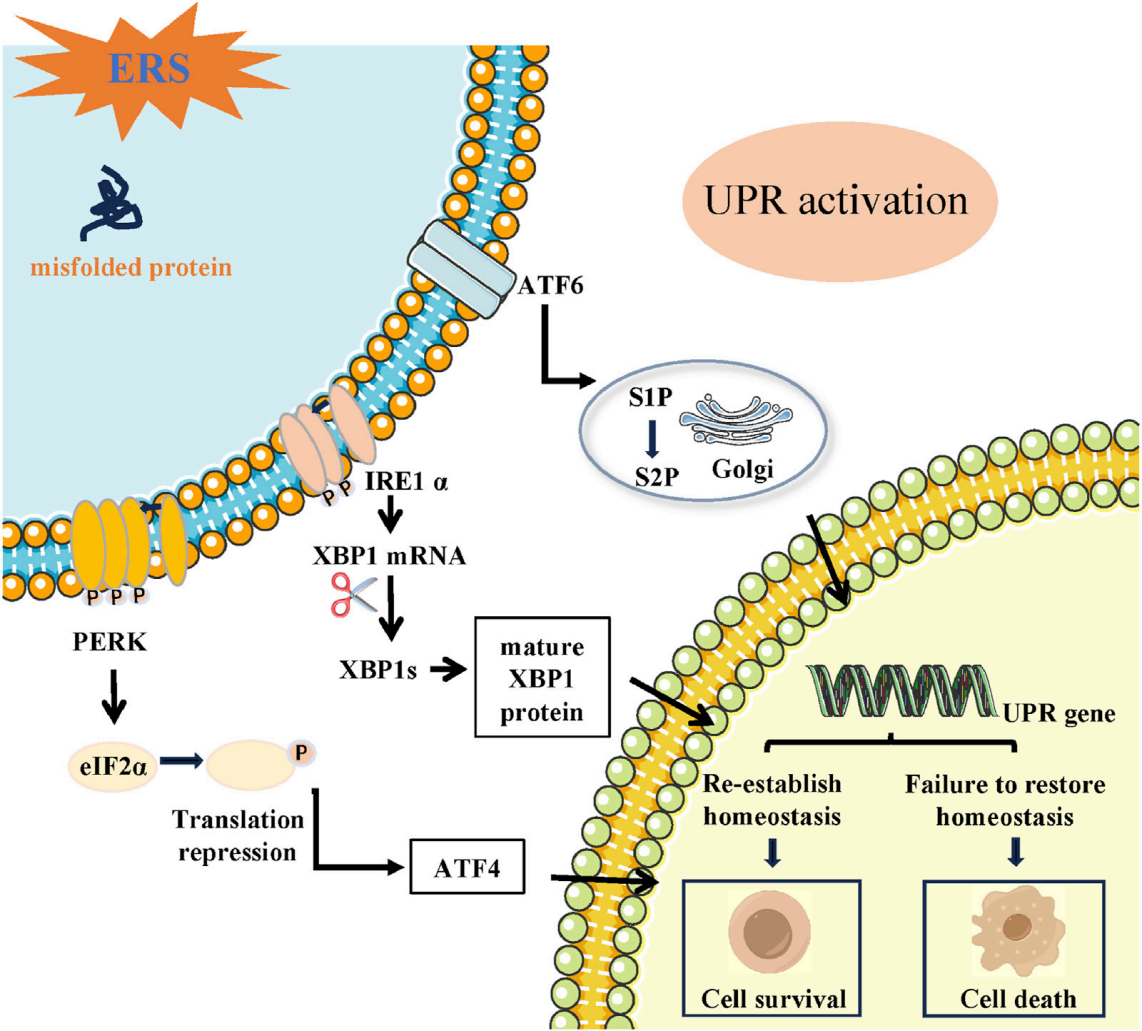
Schematic of the three signaling pathways during UPR induced by ERS (IRE1α, ATF6, and PERK). ER transmembrane proteins mainly include IRE1, PERK and ATF6. Following ERS, unfolded proteins are accumulated in the ER lumen. The three branches from three ER transmembrane stress sensors are activated that shape the UPR. PERK dimerizes, gets phosphorylated, and activated causing phosphorylation of eIF2α. Activated eIF2α suppress global protein synthesis inhibition but stimulates the translation of ATF4. IRE1α oligomerizes and auto-phosphorylates and its RNase domain becomes active and targets XBP1 mRNA, leading to the expression of the spliced form of XBP1 (XBP1s). Activated ATF6 translocate to the Golgi and is sequentially cleaved by the Golgi-resident site-1 and site-2 proteases (S1P and S2P), thereby releasing cleaved ATF6. The transcription factors ATF4, XBP1 and ATF6 enter the nucleus and regulates downstream UPR gene, resulting in cell survival or death.

Typically, UPR consists of three parallel routes triggered by distinct ERS transducers including protein kinase RNA (PKR) -like ER kinase (PERK), activating transcription factor 6 (ATF6), inositol-requiring enzyme 1α (IRE1α) (Maekawa and Inagi, 2017). During a stable condition, the three ER detectors (PERK, IRE1α, and ATF6) remain dormant through their interaction with the ER protein chaperone, referred to as glucose-regulated protein 78 kDa (GRP78) (Tsai et al., 2018). During ERS, GRP78 selectively attaches to misfolded proteins and is separated from PERK, IRE1α, and ATF6, leading to the initiation of UPR pathway (Hakim et al., 2015) (Figure 1).

### 2.1 The IRE1α signaling pathway

IRE1α, being the most conserved protein responsible for sensing UPR, exhibits both protein kinase and endoribonuclease (RNase) activities (Nishitoh et al., 2002; Takeda et al., 2019). ERS induces IRE1α kinase autophosphorylation, facilitating the cleaving of X-box binding protein 1 (XBP1) mRNA and generating spliced XBP1 (XBP1s) and un-spliced XBP1 (XBP1u) (Yoshida et al., 2001). The XBP1s splice variant activates the transcription of a cluster of genes responsible for regulating the ER’s quality control mechanisms, while the un-spliced form inhibits the expression of these genes (Apostolou et al., 2019; Takeda et al., 2019). Furthermore, IRE1α might possess functionalities that are not reliant on XBP1 mRNA splicing. IRE1α can cleave mRNAs to relieve protein load by a regulated IRE1α-dependent decay (RIDD) process (Tam et al., 2014).

### 2.2 The PERK signaling pathway

As a significant transducer of the ERS response, PERK signaling is activated by luminal-domain mediated homodimerization and autophosphorylation in response to ERS (Balsa et al., 2019). By directly phosphorylating eukaryotic initiation factor 2α (eIF2α), active PERK halts protein synthesis and decreases the load of ER cargo, creating an opportunity for enhanced protein folding. At the same time, the phosphorylation of eIF2α also aids in the translation of the transcription factor 4 (ATF4), a crucial outcome of UPR that controls the expression of multiple genes related to antioxidant response, apoptosis, autophagy, and the synthesis and transportation of amino acids (Harding et al., 2003; Dey et al., 2015; Carletti et al., 2019; Tameire et al., 2019; Lin et al., 2021). Specifically, the C/EBP homologous protein (CHOP), which is a transcriptional factor involved in cell death, rises in levels when PERK phosphorylates eIF2α (Kopp et al., 2019; Zhang et al., 2020).

### 2.3 The ATF6 signaling pathway

During periods of inactivity, ATF6, a transmembrane protein of type II, remains confined within the ER membrane (Ye et al., 2000; Bright et al., 2018). Upon encountering ERS, ATF6 is translocated to the Golgi, where it undergoes cleavage by serine site-1 protease (S1P) and metalloprotease site-2 protease (S2P) (Ye et al., 2000; Shen and Prywes, 2004). After being cleaved, ATF6 moves to the nucleus and acts as a transcription factor that regulates the expression of genes related to the UPR, as stated in reference and regulate the expression of downstream genes related to the UPR. (Haze et al., 1999).

## 3 ERS: the connection with biological response

However, UPR has its “dual nature.” The pro-survival protection effects of UPR under moderate ERS also coverts to pro-death program when ERS is prolonged and uncontrollable (Chen B. L. et al., 2015).

### 3.1 Apoptosis

Apoptosis is the classically best-understood cell death processes that denotes a specific caspase-dependent programmed cellular death (Seifert and Miller, 2017). Apoptosis is characterized by the liberation of cytochrome c from mitochondria, which is controlled by an equilibrium between pro-apoptotic and anti-apoptotic proteins belonging to the BCL2 group, as well as initiator caspases (caspase-8, -9, and -10) and executioner caspases (caspase-3, -6, and -7) (Brentnall et al., 2013). These occurrences are referred to as indicators of apoptosis and frequently utilized to determine the specific pathway of cell death (Bertheloot et al., 2021). Apoptosis takes place via two pathways: death-receptor dependent apoptosis, which is extrinsic, and mitochondrial dependent apoptosis, which is intrinsic. Initiation of the extrinsic pathway occurs when death receptors on the cell surface are activated. Death receptors that promote apoptosis include TNF, Fas, and TNF-related apoptosis-inducing ligand (TRAIL) receptors, specifically death receptor 4 (DR4) and DR5. The activation of these death receptors triggers caspase-8, which starts the process of proteolytic cell death mediated by executioner caspases (Chuang et al., 2016; Trejo-Solís et al., 2018). The intrinsic pathway consists of the mitochondrial pathway and the intrinsic endoplasmic reticulum pathway. Disruption or imbalance in intracellular balance caused by harmful substances or DNA damage activates pro-apoptotic BH3-only proteins, leading to the creation of BAX and BAK pores. These pores are responsible for permeabilizing the outer membrane of mitochondria, a process known as mitochondrial outer membrane permeabilization (MOMP). The release of proteins in the intermembrane space, such as cytochrome c, is facilitated by MOMP, leading to the activation of caspase and the occurrence of apoptosis (Shamas-Din et al., 2013; Xue et al., 2023). Caspase-12, functioning as an ER-resident caspase, is selectively activated upon the occurrence of ERS, leading to the activation of caspase-9/-3 and ultimately initiating apoptosis (Iurlaro and Muñoz-Pinedo, 2016).

**FIGURE 2:**
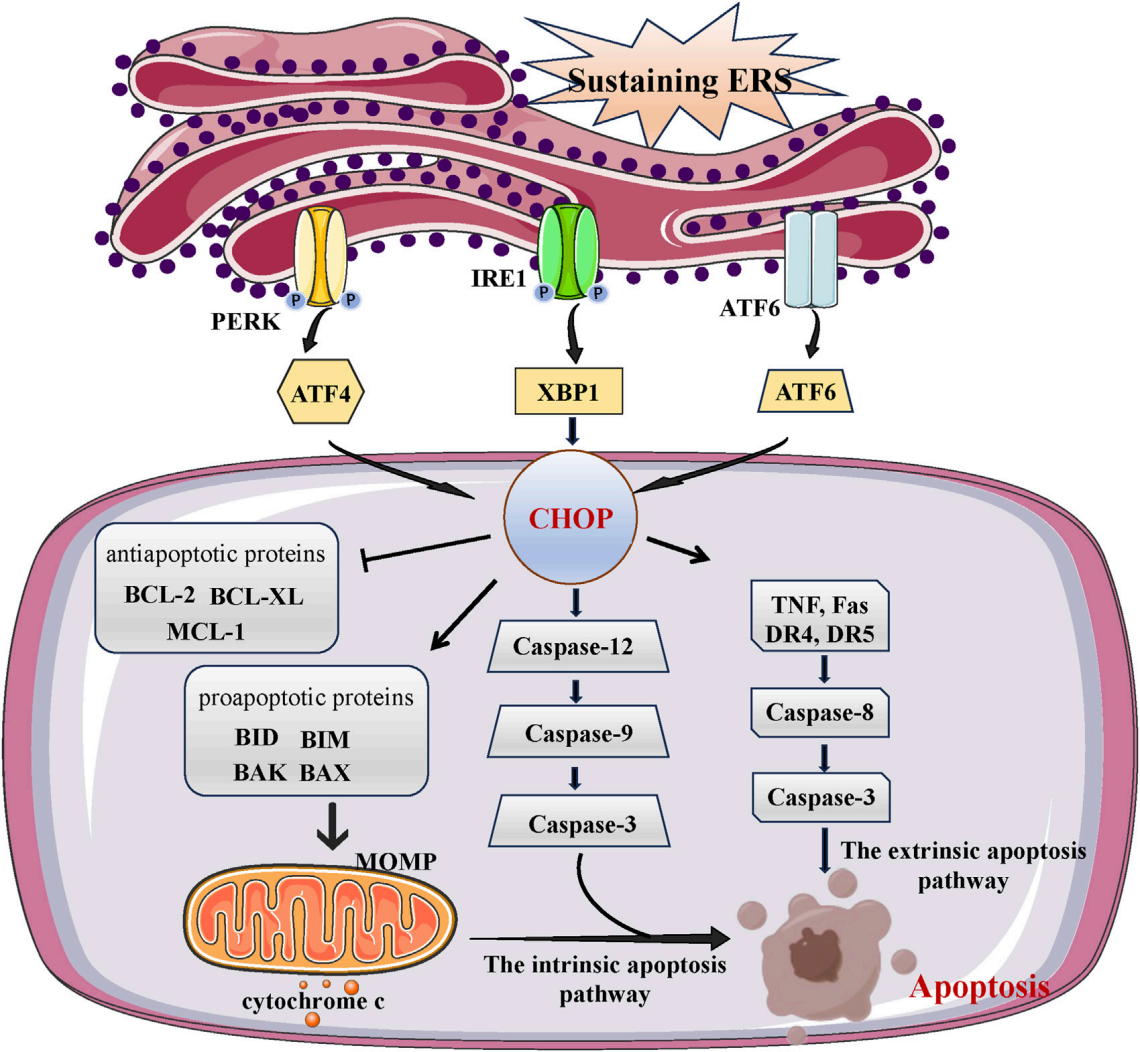
The link between ERS and apoptosis. Under overwhelming conditions of ERS, the UPR induces the activation of both intrinsic and extrinsic apoptosis pathways. Death receptor-mediated extrinsic apoptosis Receptor-mediated pathways are initiated by death-ligands that bind to their specific death receptors, which include TNF, Fas and TNF-related apoptosis-inducing ligand (TRAIL) receptor (DR4, DR5). The intrinsic pathway of apoptosis is comprised with mitochondria pathway and ERS pathway. Mitochondrial apoptosis pathway is induced by BCL-2 family proteins (including anti-apoptosis proteins and pro-apoptosis proteins), while ERS apoptosis pathway is induced by caspase-8. After a series of reactions, intrinsic and extrinsic apoptosis pathways eventually trigger cell apoptosis.

The ERS pathway recently is described as another important apoptosis pathway (Boyce and Yuan, 2006). Based on the literature, CHOP promoter can be bound and its transcription can be activated by all three branches (ATF6, IRE1, ATF4) in response to excessive ERS. CHOP is characterized as a significant facilitator of the ERS-triggered cell death pathway. Cells lacking CHOP exhibit resistance to apoptosis induced by ERS (Oyadomari and Mori, 2004; Malhi et al., 2013; Nam et al., 2015). CHOP activation leads to the irreversible promotion of caspase-12 entry into the cytoplasm, initiating the caspase cascade and ultimately inducing ERS-induced apoptosis (Bernales et al., 2006; Lakshmanan et al., 2011). Research has discovered that CHOP has the ability to trigger cell death through both the extrinsic death receptor and intrinsic mitochondria-dependent pathway, as stated in a study (Oyadomari and Mori, 2004). By binding to the death receptor pathway and increasing the levels of DR4 and DR5, CHOP has the ability to trigger apoptosis. This activation of caspase-8 ultimately leads to cell death (Shamas-Din et al., 2013; Hu et al., 2018). However, CHOP has the ability to control the expression of numerous genes involved in apoptosis, such as those responsible for producing BCL2 family proteins, GADD34, and DOCs (Tabas and Ron, 2011). There are two types of proteins in the BCL2 protein family: anti-apoptotic proteins and pro-apoptotic proteins. The main proteins involved in preventing apoptosis are BCL2, BCL-XL, and MCL-1. Pro-death proteins are composed of proteins with multiple domains and proteins with only the BH3 domain (Ding et al., 2017). Proteins with multiple domains include BAX and BAK, whereas proteins with only the BH3 domain include BID, BIM, BAD, NOXA, and PUMA (Chen et al., 2014; Fitzsimmons et al., 2020). The main function of BH3-only proteins is to control cell apoptosis by either suppressing the production of the BCL2 anti-apoptotic protein or enhancing the production of multidomain proteins like BAX. CHOP has the ability to decrease the expressions of BCL2, BCL-XL, and MCL-1 while increasing the expression of BIM, resulting in elevated expression of BAK and BAX (Tsukano et al., 2010; Iurlaro and Muñoz-Pinedo, 2016). Moreover, mitochondria permeabilization facilitates the liberation of apoptotic elements like cytochrome c and apoptosis-inducing factor (AIF), ultimately resulting in cellular apoptosis (Castera et al., 2009) (Figure 2).

Activation of caspase-3 and BAX proteins could induced cell apoptosis by increasing CHOP in the Sprague-Dawley rat model of IRI through excessive ERS (Tan et al., 2020a). During a separate experiment conducted on live rats and in laboratory cell models called NRK-52E, the levels of ERS response proteins such as CHOP, GRP78, XBP1, and ATF6 have showed a notable increase in response to IRI. Following IRI, there was a significant increase in the levels of BAX and cleaved caspase-3, accompanied by a notable decrease in BCL2 expression. The findings from all experiments suggest that ERS stimulates cell death by activating the caspase cascade induced by CHOP (Tan et al., 2020b).

### 3.2 Autophagy

The process of autophagy is a well-preserved mechanism that transports cellular components to lysosomes for breakdown (Ohsumi, 2014). Autophagy functions as a crucial cellular recycling process that safeguards cells from harm caused by impaired organelles, faulty or clumped proteins, lack of nutrients, oxidative damage, and low oxygen levels (Levine and Kroemer, 2008; Levine et al., 2011; Kim and Lee, 2014). Autophagy encompasses macroautophagy, microautophagy, and chaperone-mediated autophagy, with macroautophagy being the extensively researched form (referred to as “autophagy” hereafter) (Tusco et al., 2017). Autophagy consists of multiple sequential processes, including induction (initiation), the formation of autophagosomes, the fusion of autophagosomes, and degradation (Zhao Y. G. et al., 2021). Specifically, autophagy is initiated by the formation of a phagophore, a double membrane structure that takes the shape of a crescent. This phagophore expands and surrounds cellular components from the cytoplasm, which are then broken down and transformed into a double membrane autophagosome. Autophagosomes combine with lysosomes to create autolysosomes, in which the enclosed contents are broken down by the action of lysosomal enzymes. The complete and ongoing process is known as autophagy flux. In the process of autophagy, a variety of autophagy related proteins regulate and control the different stages of autophagy formation. Microtubule-associated protein 1 light chain 3 (LC3B) is a key protein and throughout autophagy process. LC3B protein is converted from LC3B I to LC3B II when autophagy, LC3B II associates with the membrane of autophagosomes and participates in following autophagy process (Kabeya et al., 2000; Delcour et al., 2019). p62/SQSTM1(p62), a selective autophagic substrate, connects with LC3B and targets ubiquitinated protein to mature autophagosomes for degradation through ubiquitin signaling pathway (Glick et al., 2010). p62 accumulates when the lysosomal degradation of the autophagosome is blocked, Therefore, the level of p62 is used to measure the ability of autophagy clearance and degradation (Klionsky et al., 2021). Therefore, LC3B and p62 are two marker protein that generally indicate the induction of autophagy (Yoshii and Mizushima, 2017).

Autophagy and ERS have been found to be interconnected in recent research, exhibiting common characteristics such as safeguarding cells by alleviating stress and triggering cell demise in challenging circumstances. ERS could promote autophagy and *vice versa*. On the one hand, ERS acts as an autophagy inducer. Moderate ERS induces autophagy to help remove unfolded or misfolded proteins, which alleviate ERS and promote cell survival (Fernández et al., 2015). However, under certain pathological conditions, ERS becomes dysregulated, leading to compromised autophagy. In Huntington’s disease, ERS can inhibit autophagy flux through IRE1 kinase activity, leading to neuronal degeneration and disease progression (Lee et al., 2012). In the familial amyotrophic lateral sclerosis (ALS) model, XBP1 deficiency increases the expression of several genes that positively regulate autophagy, leading to upregulation of basal autophagy (Balsari et al., 1985). The difference in the function of ERS in autophagy flow could arise due to varying sensitivity of cell types to ER stress or the duration of ERS, whether it is acute or chronic stress.

**FIGURE 3:**
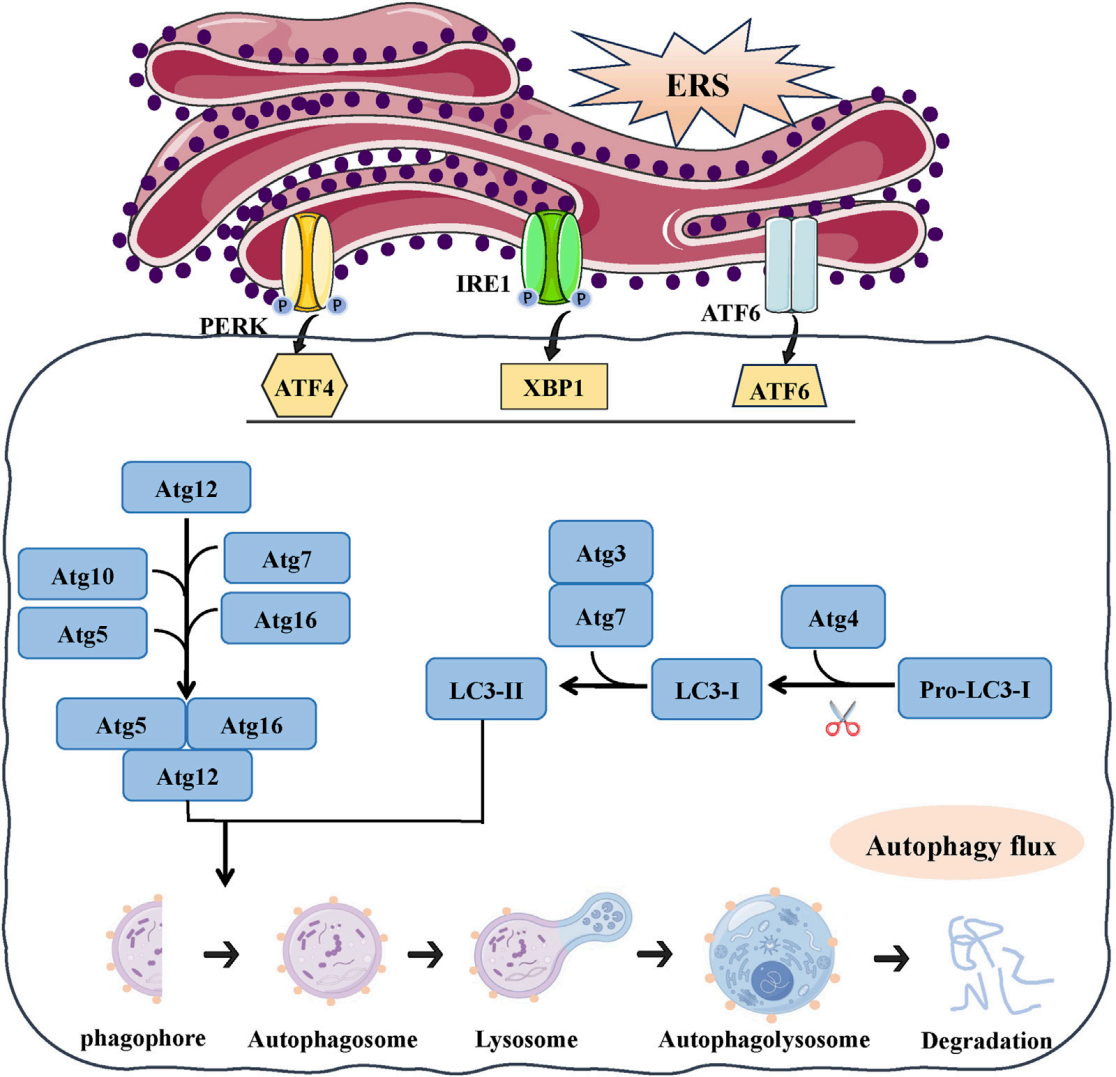
The link between ERS and autophagy. ERS could activate autophagy, the UPR can regulate expression of autophagy gene (Atg3, Atg5, Atg7, Atg10, LC3B, P62) and autophagosome formation. Autophagy begins with autophagosome formation, LC3B localizes on the surface of autophagic vesicle membrane and participates in autophagosome formation. With autophagy proceeds, autophagosome fuse with lysosomes generates an autophagolysosome and results in degradation.

Three UPR pathway regulate autophagy in different ways during ERS (Yu et al., 2021). The IRE1 pathway is required for uncontrolled autophagy under ERS. When treated with ERS agents, no autophagosome formation has been observed in an IRE1 knockout mutant (Liu et al., 2012; Yang et al., 2016). According to a recent study, the activation of autophagy via the IRE1/XBP1 pathway can improve the elimination of misfolded proteins by ER-associated degradation. This presents a hopeful treatment strategy for reducing ER stress and tackling cardiovascular disorders (Wang et al., 2016). The ATF6 and PERK pathway appears controversial in regulating the autophagic process. According to a study conducted in 2012, autophagy does not involve either ATF6 or PERK signaling. Cells exposed to XBP1 siRNA exhibited characteristics of autophagic cell demise, while cells exposed to ATF6 and PERK siRNA exhibited characteristics of programmed cell death (Pehar et al., 2012). Ongoing research indicates that the activation of autophagy can be induced by the PERK pathway and the ATF6 pathway, either for the purpose of restoring ER homeostasis or promoting programmed cell death. During extended periods of stress, the activation of a group of autophagy genes (Atg3, Atg5, Atg7, Atg10, LC3B) is facilitated by the binding of p62 with ERS, ATF4, and CHOP transcription factors, leading to the promotion of cell survival (B’Chir et al., 2013) (Figure 3).

Meanwhile, the ATF6 pathway is considered necessary for the induction of autophagy mediated by the Peste des petits ruminants virus (PPRV) (Zhang R. et al., 2022). According to a study conducted in 2016, the ERS eIF2α/ATF4 pathway plays a role in cadmium-induced kidney autophagy and injury by causing COX2 overexpression (Luo et al., 2016). The activation of the ERS/ATF4 pathway leads to an increase in COX2 expression, which in turn contributes to the development of kidney autophagy and damage in lupus nephritis (Jin et al., 2018).

### 3.3 Ferroptosis

AKI is significantly influenced by the build-up of lipid peroxides that rely on iron and is a crucial factor in its advancement and growth. The main factors contributing to the ferroptosis process are the imbalance of the antioxidant system, disturbance in iron metabolism, and the buildup of lipid peroxidation (Zhang C. et al., 2022).

The system xc-/glutathione (GSH)/glutathione peroxidase 4 (GPX4) pathway has a vital function in controlling cellular redox balance and impacting the result of ferroptosis within the antioxidant mechanisms context (Yang and Stockwell, 2016). System xc- operates as an internal antioxidant system in this pathway, consisting of a heterodimer made up of a light chain (xCT, SLC7A11) and a heavy chain (4F2hc, SLC3A2). GPX4 promotes the connection between GSH and ROS, leading to the conversion of harmful lipid hydroperoxides (PUFA‐OOH) into harmless lipid alcohols (PUFA‐OH), thereby alleviating oxidative stress within cells (Muri et al., 2019). The system xc-typically has a crucial function in controlling levels of glutamate outside the cell by exporting glutamate while simultaneously importing cysteine to aid in the production of intracellular GSH. The GSH reservoir functions as the reducing agent utilized by GPX4 to combat the advancement of ferroptosis (Stockwell and Jiang, 2020). Therefore, upregulation of the system xc-may be a potential approach to mitigate various disorders and enhance the cell’s antioxidant defense by increasing GSH synthesis. ROS levels are elevated due to the Fenton reaction caused by surplus iron resulting from enhanced transferrin transportation and ferritinophagy, and this excess ROS is counteracted by iron in reverse (Arefieva et al., 2021). During the transferrin transport process, the transferrin receptor (TFR) binds to di-ferric transferrin (TF) and promotes Fe^3+^ transport. STEAP3 deoxidized iron in Fe^3+^ form to iron in Fe^2+^ by acting as an iron oxide reductase. Afterwards, ferrous ion (Fe^2+^) is carried to the cytosol through the action of divalent metal transporter 1 (DMT1), allowing it to join the labile iron pool (LIP) within the cytosol. The ferritin, which is made up of 24 subunits of heavy (FTH1) and light (FTL) isoforms, can store this iron that has been internalized. Ultimately, iron export is facilitated by ferroportin (FPN) (Han et al., 2023). Ferritinophagy refers to the degradation of ferritin, which is facilitated by the nuclear receptor coactivator NCOA4 (Li et al., 2021). NCOA4 directly identifies and attaches to FTH1, subsequently transporting iron-bound ferritin to autophagosomes to be degraded in lysosomes and released from iron (Fang et al., 2021). Transferrin transport and ferritinophagy are important for maintaining cellular iron homeostasis. Also, abnormal lipid peroxidation and lethal lipid peroxides (LPOs) accumulated is also believed to play pivotal part in ferroptosis and effect cell vulnerability to ferroptosis (Zhang C. et al., 2022). Polyunsaturated fatty acids (PUFAs), including linoleic acid (LA), linolenic acid (LNA), and arachidonic acid (AA), are highly susceptible to peroxidation, leading to the formation of LPO and facilitating the generation of ferroptosis (Doll et al., 2017). Ferroptosis heavily relies on Acyl-CoA synthetase long-chain family member 4 (ACSL4) and lysophosphatidylcholine acyltransferase 3 (LPCAT3) as crucial catalysts. ACSL4 facilitates the interaction of coenzyme A with long-chain PUFAs, resulting in the formation of long-chain fatty acyl-CoA esters. The LPCAT3 enzyme re-esterifies these items into phospholipids, thereby improving the integration of long-chain PUFAs into lipids and membranes (Dixon et al., 2015). The action of lipoxygenases (LOX) on PUFAs results in the formation of PUFA‐OOH, leading to the buildup of lipid peroxides (LPO) that are accountable for the generation of compounds resulting from the breakdown of lipid peroxides (Mao et al., 2020).

**FIGURE 4:**
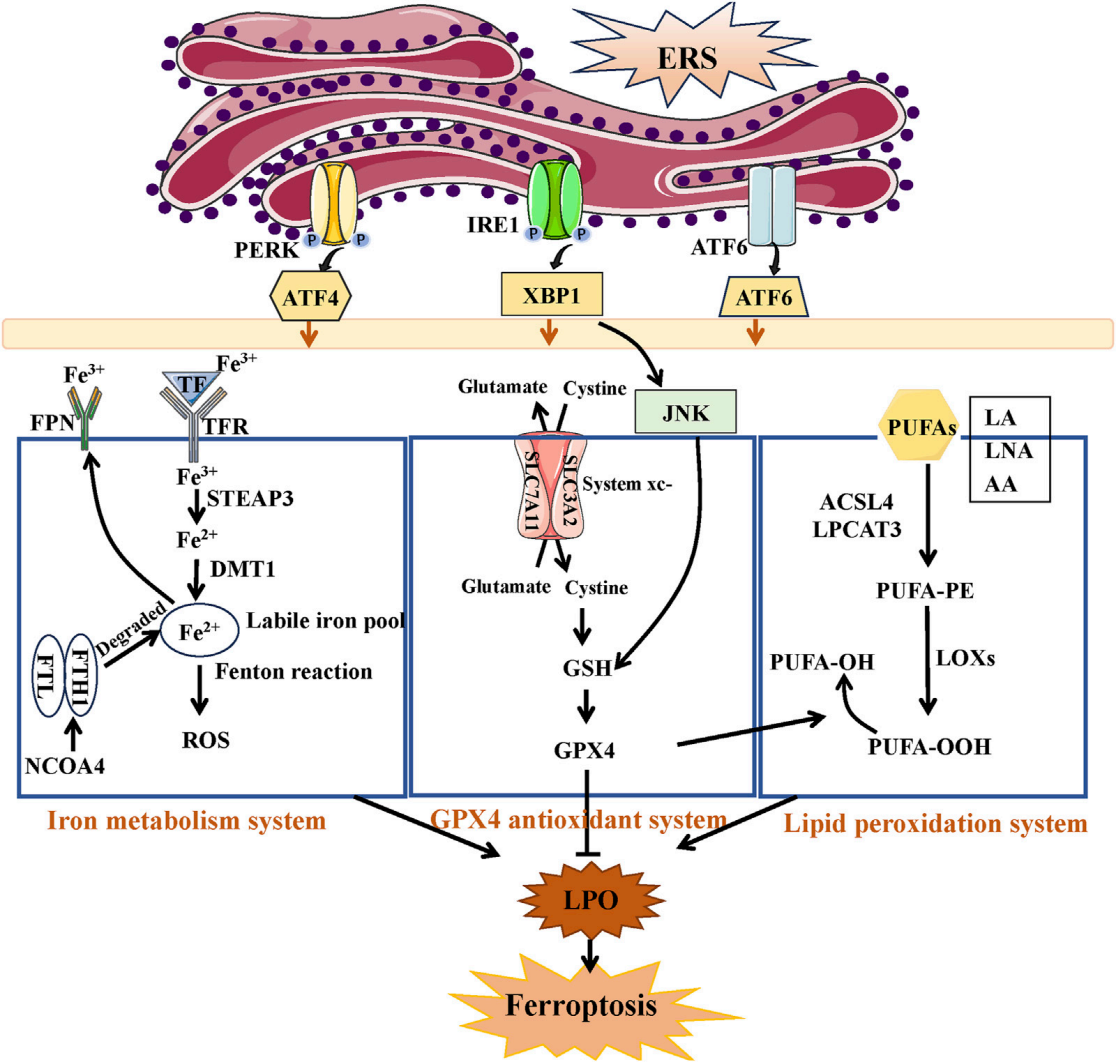
The link between ERS and ferroptosis. Under ERS, the three branches of UPR can regulate ferroptosis through iron metabolism system, GPX4 antioxidant system, and lipid peroxidation system. The accumulation of Fe^2+^ and PUFA-OOH or the inactivation of GPX4 antioxidant system all induce the production of lipid peroxides (LPO), resulted in ferroptosis.

Figure 4 illustrates that there is an increasing amount of evidence that has provided insight into the interaction known as “crosstalk” between ERS and ferroptosis (Chen et al., 2017; Li et al., 2020). The interaction between the IRE1 pathway and ferroptosis is confirmed in AKI induced by IRI. Mitigating ferroptosis may offer protection against renal injury by inhibiting the IRE1 pathway (Liang et al., 2022). Ferroptosis could be triggered via the PERK/eIF2α/ATF4/CHOP pathway in AKI induced by cadmium, and the suppression of endoplasmic reticulum stress (ERS) alleviated cadmium-induced ferroptosis (Zhao C. et al., 2021).

### 3.4 Pyroptosis

The definition of pyroptosis has been first suggested by D’Souza in 2001 (Ma et al., 2021). Gasdermin D (GSDMD) is responsible for pyroptosis, which is a form of programmed cell death characterized by inflammation. The main morphological features include cell enlargement and membrane breakage, along with the discharge of pro-inflammatory substances (Man et al., 2017), leading to a series of escalated inflammatory reactions and activated immune responses (Fink and Cookson, 2007; Shi et al., 2015). Pyroptosis, a form of cell death, can occur through two primary molecular mechanisms: the caspase-1-dependent canonical pathway and the caspase-4/5/11-dependent non-canonical pathway. Pyroptosis is initiated in the canonical pathway through the activation of inflammasomes, primarily the NOD-like receptor thermal protein (NLR) family (NLRP1, NLRC4, NLRP3) and the pyrin protein families. Inflammatory reactions are caused by the activation and cleavage of pro-caspase-1 by these inflammasomes, resulting in the formation of active caspase-1. This active caspase-1 then cleaves GSDMD to produce the N-terminal form (GSDMD-N), which possesses pore-forming activity. Ultimately, the secretion of pro-inflammatory cytokines interleukin-1β (IL-1β) and IL-18 occurs as a consequence, leading to the inflammatory reactions (Rosenzweig and Chopra, 2013). In contrast to the canonical pyroptosis pathway, the non-canonical pyroptosis pathway is triggered by caspase-4/5/11. This leads to the direct cleavage of GSDMD, resulting in the induction of cell pyroptosis.

**FIGURE 5:**
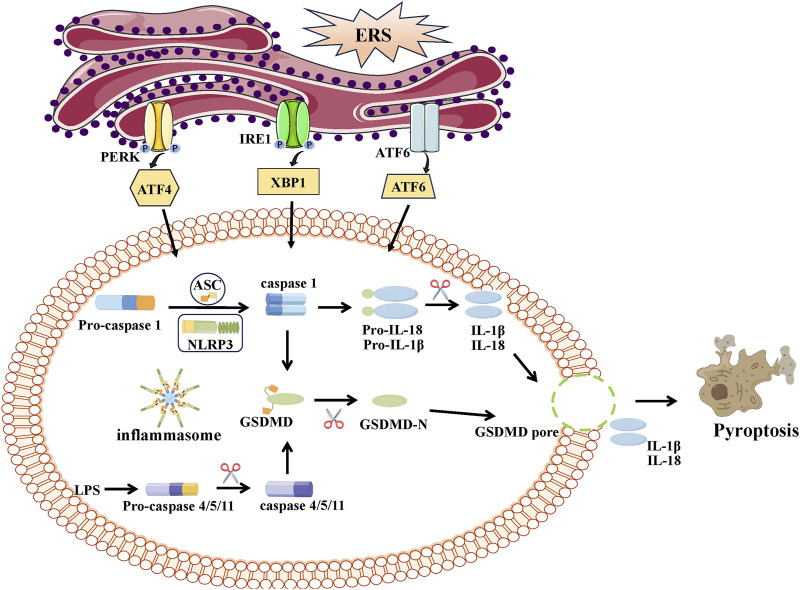
The link between ERS and pyroptosis. Pyroptosis can be categorized in two distinct ways: caspase-1-dependent classical inflammasome signaling and caspase-4/5/11-dependent nonclassical inflammasome signaling. Activated caspase-1 and caspases-4/5/11 cleave GSDMD into a N-terminal fragment of GSDMD (GSDMD-N) and promote maturation of IL-1β and IL-18. GSDMD-N can bind and form pores at cell membrane, leading to the release pro-inflammatory cytokines (IL-1β, IL-18) and the progress of inflammation and pyroptosis.

ERS activates inflammasome activation (Vanaja et al., 2015; Han et al., 2018; Zhou et al., 2020) and promotes pyroptosis (Nie et al., 2023; Yan et al., 2023). ERS has the potential to greatly induce the activation of NLRP3/caspase-1 and enhance the expression of genes associated with pyroptosis. Suppression of ATF4 signaling significantly reduces the levels of NLRP3 inflammasome constituents (Vanaja et al., 2015). Maintaining the stability of the ER seems crucial in preventing excessive activation of the NLRP3 inflammasome, decreasing caspase-1-mediated pyroptosis, and enhancing sepsis results (Wang et al., 2021) (Figure 5).

In a recent study, a connection between ERS and pyroptosis was successfully established (Li et al., 2022). All three UPRs contributes to ERS-induced pyroptosis. Initially, a correlation is noted between PERK/ATF4 communication and caspase-1-induced pyroptosis. In the sepsis mouse model, the activation of the PERK/ATF4/CHOP signaling pathway trigger NLRP3/caspase-1-mediated pyroptosis and the generation of pro-inflammatory cytokines, leading to elevated mortality rates among septic mice. By reducing the expression of the PERK/ATF4/CHOP signaling pathway, the NLRP3 inflammasome’s hyperactivation and caspase-1-dependent pyroptosis are inhibited, which improving sepsis outcomes (Wang et al., 2021). The coronavirus disease 2019 (COVID-19) pandemic is caused by SARS-CoV-2, a highly transmissible virus. During SARS-CoV-2 infection, a study indicates that there is a communication link between endoplasmic reticulum stress (ERS) and the inflammatory response (pyroptosis) in lung epithelial cells, which occurs through the activation of PERK. Inhibition of PERK can mitigate inflammation, while pyroptosis leads to the suppression of all inflammatory cytokines, as well as the downregulation of NF-κB and decreased protein expression of IL-1β and IL-18 precursors in cellular extracts (Keramidas et al., 2023). Additionally, research has documented the impact of the IRE1/XBP1 pathway on ERS-induced pyroptosis (Cao et al., 2023). A study has shown that the activation of IRE1α is markedly increased in a rat model experiencing traumatic brain injury. Inhibition of the IRE1α signaling pathway may enhance neuronal NLRP1-induced pyroptotic cell death (Vanaja et al., 2015). The activation of the UPR pathway induced by IRE1α could potentially contribute to inflammatory response and damage to the brain in a rat model of neonatal hypoxic-ischemic encephalopathy (Chen et al., 2018). Inhibition of IRE1α alleviates neuronal pyroptosis and enhances neurobehavioral outcomes via the miR-125/NLRP1/caspase-1 signaling pathway (Huang et al., 2020). A 2020 study has revealed that increased oxidative stress and ERS promote pyroptosis and the anticancer effect, which may be exploited to develop new cancer prevention and therapies (Le et al., 2020). Moreover, the ATF6 pathway plays a crucial function in regulating pyroptosis. In ERS induced by silver nanoparticles, ATF6 signaling is crucial for induction of pyroptosis. By inhibiting the processing of ATF6, the activation of NLRP3 is suppressed, leading to a decrease in caspase-1 activity, secretion of IL-1β, and the proportion of deceased cells. The activation of this inflammasome and the occurrence of pyroptosis could be induced by ATF6 signaling, potentially functioning as a molecular switch (Simard et al., 2015). Markers specific to ERS such as GRP78, CHOP, and caspase-12, are triggered following renal IRI, leading to elevated levels of proteins associated with pyroptosis (NLRP3, ASC, caspase-1, GSDMD-N, caspase-11, and IL-1β). ERS inhibition induced by naringenin treatment markedly restricts pyroptosis-associated proteins and mitigated renal IRI (Zhang B. et al., 2022).

## 4 ERS: the role in several models of AKI

**FIGURE 6:**
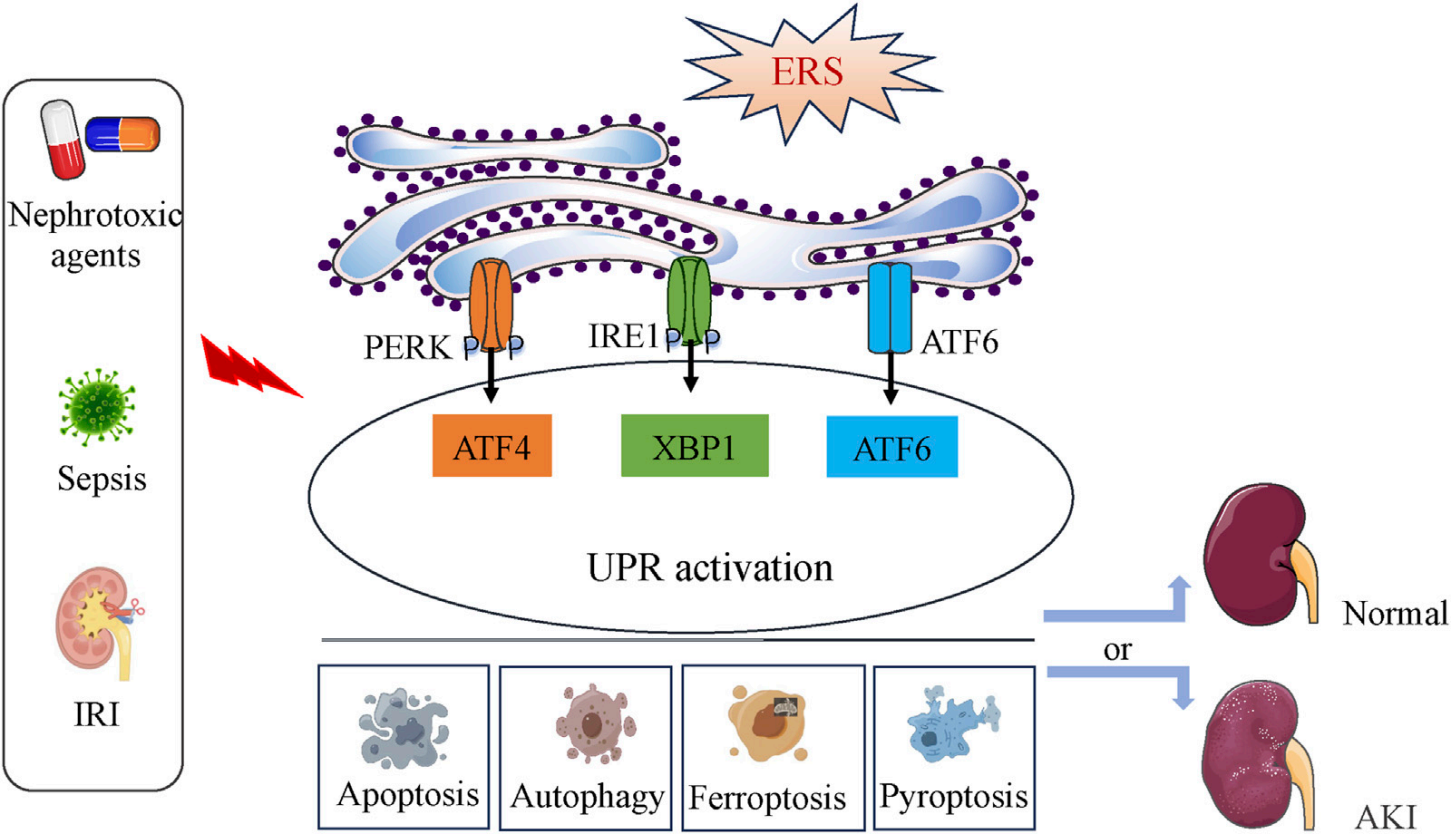
The dichotomous effect of ERS in AKI. ERS and the activation of the UPR pathway occur during IRI, nephrotoxic agents or sepsis induction. Depending on the intensity of the ERS, UPR activation can directly affect the occurrence and progress of apoptosis, autophagy, ferroptosis and pyoptosis, then lead to cell survival or death, finally determine the development of AKI.

ERS play dual roles in AKI contributing to the cell fate in kidney (Figure 6).

### 4.1 ERS in renal ischemia-reperfusion injury

IRI frequently happens after infection, trauma, significant cardiovascular surgery, and kidney transplantation (Pefanis et al., 2019). Renal IRI is one of the leading causes of clinical AKI, a common pathophysiological phenomenon in clinical settings with high morbidity and mortality (Ricci et al., 2008; Coca et al., 2009). Studies conducted recently have suggested that ERS has a part to play in the induction of IRI, particularly in the renal tissue. The current literature depicts ERS as a two-sided weapon. On the other hand, a moderate ERS could potentially provide protection against renal IRI. Multiple research studies have shown that the activation of UPR, through the use of UPR inducers like tunicamycin, thapsigargin, and A23187, can potentially alleviate the extent of renal injury caused by IR (Bush et al., 1999; George et al., 2004; Prachasilchai et al., 2008). In 2015, a study also emphasizes the advantageous effect of ERS-triggered autophagy on renal IRI (Chandrika et al., 2015). Activation of ERS signaling induces autophagy, resulting in cyto-protection against oxidative damage and chemical-induced hypoxia, ultimately enhancing renal IRI. UPR activation is a new opportunity for pharmacological intervention against renal IRI (Prachasilchai et al., 2008; Prachasilchai et al., 2009).

However, the overstatement of ERS plays a role in the advancement of renal IRI (Li et al., 2023). The upregulation of ERS-related proteins like GRP78 and CHOP have been observed in mice models of renal IRI. Inhibition of ERS activation could alleviate cell apoptosis and IR-induced renal injury (Liu et al., 2019; Chen et al., 2022). According to a study conducted in 2012, proteins associated with endoplasmic reticulum stress (ERS) such as GRP78, PERK, ATF4, and XBP1 is increased during renal ischemia-reperfusion injury (IRI) and played a role in the decline of renal function (Mahfoudh-Boussaid et al., 2012). ER-mitochondria tethering complexes in ERS transmit stress signals from the ER to mitochondria and oversee mitochondrial quality control, which is essential for renal IRI (Zhao et al., 2020). The IRE1/XBP1 pathway is important in modulating ERS and mitochondrial crosstalk. XBP1 activation promotes tissue damage, and cell apoptosis disrupted mitochondria. Interfering with XBP1 suppress caspase-1-mediated mitochondrial injury and decreased the generation of mitochondrial ROS, leading to a significant enhancement in kidney function (Ni et al., 2023). The involvement of the NRF2/HO-1 pathway is noted in ERS-induced renal IRI. Renal IRI lead to the deactivation of the NRF2/HO-1 signaling pathway, along with an increased expression of GRP78, eIF2α, and CHOP protein. Activation of the NRF2/HO-1 signal limited the increase in levels of ERS-associated proteins, thereby relieving the impaired renal function and structural harm caused by IRI (Yu et al., 2022). Moreover, the involvement of connexin32 in IRI-induced AKI is crucial as it triggered the ROS/ERS/apoptosis signaling pathway. On the other hand, the absence of connexin32 lead to the elimination of ROS and the suppression of ERS, ultimately resulting in safeguarding against renal IRI (Gu et al., 2018).

### 4.2 ERS in drug nephrotoxicity

One of the significant forms of AKI is AKI caused by drugs. According a literature in 2015, the incidence of drug-related AKI reach up to 60% (Ghane Shahrbaf and Assadi, 2015). A survey conducted in multiple centers in China indicates that 71.6% of individuals who experienced AKI had previously used drugs that could potentially harm the kidneys, either before or during the occurrence of renal injury (Yang et al., 2015). A different Chinese survey conducted across the country shows that AKI is mainly associated with anti-cancer drugs (cisplatin), medications for infections, and toxic metals (Liu et al., 2021).

ERS has been linked to nephrotoxic acute kidney injury in the past few years. ERS preconditioning provides cyto-protection against nephrotoxins that are clinically relevant in renal cell lines, on the other hand. Renal cells preconditioned with ERS inducer exhibits a notable decrease in the toxicity of medically significant medications (cisplatin, gentamicin, glyoxylate, cyclosporine A, p-aminophenol) (Peyrou and Cribb, 2007; Peyrou et al., 2007). Conversely, the involvement of the ERS signaling pathway plays a crucial part in the pathogenesis of acute nephrotoxicity. The use of Cisplatin is widely acknowledged as an effective therapy for different types of malignancies (Bottone et al., 2008). However, its clinical application and therapeutic effectiveness are significantly hindered by its nephrotoxicity (Pabla and Dong, 2008). Exposure to cisplatin results in an elevation of levels of ERS-associated proteins (PERK, GRP78, caspase-12), which subsequently triggers an augmentation in ERS-induced apoptosis (Chen B. et al., 2015).

Activation of the UPR signaling pathway PERK/eIF2α/ATF4 axis is manifested and contribute to apoptosis in renal cells, resulting in renal dysfunction and tubular kidney damage (Wei et al., 2021). For anti-infectives, aminoglycosides and polymyxins are the main drugs resulting in nephrotoxicity. The expressions of caspase-12, GRP78, and CHOP are elevated in rats experiencing nephrotoxicity induced by gentamicin. The rise is accompanied by increased amounts of cleaved caspase-3 and a higher Bax/Bcl-2 ratio. The results strongly suggest that the ERS-mediated cell apoptosis signaling pathway plays a significant role in the development of nephrotoxicity (Jaikumkao et al., 2016). In colistin-induced nephrotoxicity mice, increased expression of four biomarkers (GRP78/Bip, ATF6, caspase-12, and CHOP) are observed for the endoplasmic reticulum pathway, indicating that the endoplasmic reticulum pathway is involved in colistin-induced nephrotoxicity (Dai et al., 2014). Heavy metal, cadmium and mercury activated ERS and contributes to progress of AKI. Both kidney tissues and cells exposed to cadmium induces expression of cyclooxygenase-2 (COX-2) and activation of the PERKeIF2α/ATF4 pathway associated with ERS. In particular, when ERS is partially inhibited, excessive expression of COX-2 is mitigated and autophagy is alleviated. These observations strongly indicate that ERS plays a crucial role in cadmium-induced autophagy and the subsequent development of AKI (Luo et al., 2016). In cases of mercury-induced AKI, the PERK/eIF2α branch initially activates within the first 48 h, showing a protective but insufficient response. However, prolonged activation of the PERK/eIF2α branch subsequently triggers the activation of the ATF4, ATF6, and IRE1α pathways, promoting the activation of caspases-12 and -3. These cascading events contribute to the death of tubular and glomerular cells. This underscores the pivotal role of ERS in developing mercury-induced AKI (Rojas-Franco et al., 2019). Furthermore, some chemical-induced nephrotoxicity has been considered related to ERS. Polystyrene microplastics (PSMPs), which are one of the most prevalent plastic pollutants, are recognized as sources of food contamination that are widespread and pose a significant risk to human health. The kidneys of rats are potentially affected by oxidative stress, leading to the induction of ERS and subsequent promotion of renal cell apoptosis as a result of exposure to PSMPs (Wang et al., 2022).

### 4.3 ERS in sepsis-induced AKI

Sepsis is an immune system reaction to infection that causes inflammation throughout the body. Sepsis, a major concern for public health, has emerged as the primary reason for illness and death in humans, with the annual mortality rate from sepsis-related causes showing a consistent rise (Enkhbaatar et al., 2008). AKI is a prevalent and grave consequence of sepsis, affecting around 51%–64% of individuals diagnosed with sepsis (Uchino et al., 2005; Gómez and Kellum, 2016). Nevertheless, the precise mechanisms responsible for sepsis-induced AKI are still unclear.

Numerous researches have suggested that ERS becomes active in various types of cells following microbial infection, playing a role in regulating inflammation (Xie et al., 2022). Sepsis is associated with significant activation of ERS pathway, which in turn contributes to the development of sepsis-induced AKI (Crouser, 2016). The kidney showed a robust ERS response after receiving an intraperitoneal injection of lipopolysaccharide (LPS). This was indicated by the activation of ATF4/CHOP/caspase-12 signaling and upregulation of GRP78. Md Jamal Uddin’s study (Uddin et al., 2018; Uddin et al., 2020) also indicated that activated IRE1α-JNK signaling mediated ERS may contributed to LPS-treated AKI in mice. These findings suggest that the ERS-related apoptosis process participates in septic AKI (Esposito et al., 2013; Jia et al., 2018). XBP1, which is an essential element of the ERS-activated pathways, exhibited an elevation in the kidneys of mice treated with LPS. Overexpression of XBP1 specifically in the renal tubule results in significant dilation and vacuolation of the tubule, along with the presence of injury markers Kim1 and NGAL, the pro-inflammatory molecules IL-6 and Toll-like receptor 4 (TLR4). This leads to a decline in kidney function and a mortality rate of 50% within a span of 5 days. Nonetheless, mice with XBP1 knockdown exhibited reduced levels of renal NGAL, the pro-apoptotic factor CHOP, serum creatinine, and a slight inclination towards decreased TLR4 compared to LPS-treated mice with intact XBP1. In mice with genetic XBP1 overexpression, the administration of LPS treatment resulted in a marginal elevation of NGAL and CHOP compared to mice treated with LPS alone. Hence, the distinctiveness of heightened XBP1 signaling in kidney tubules is specific to AKI caused by sepsis and plays a role in renal inflammation and damage (Ferrè et al., 2019).

## 5 Tackling ERS for AKI therapy

Due to the limited understanding of the molecular mechanisms behind AKI, there is currently a lack of effective preventive and therapeutic measures available. With knowledge of the link between ERS and AKI, regulating ERS may be a promising option for improving AKI (Table 1).

**TABLE 1:** Tackling ERS for AKI therapy.

Organs/cells	Models	Drug	Mechanism	References
LLC-PK1	cisplatin-induced nephrotoxicity	Isoliquiritigenin	Trigger protective ERS	Gómez-Sierra et al. (2020)
HK2, TCMK-1	renal IRI	TUDCA	Inhibit incurable ERS	Gao et al. (2012), Peng et al. (2015)
HK-2, Mice	renal IRI	Naringenin	Inhibit incurable ERS	Zhang et al. (2022c)
Mice, Rat	renal IRI	ROS scavenger	Inhibit incurable ERS	Wu et al. (2014), Liu et al. (2019), Zhang et al. (2021)
Rat	gentamicin-induced nephrotoxicity	Curcumin	Inhibit incurable ERS	Laorodphun et al. (2022)
Mice	sepsis-induced AKI	Forsythiaside A	Inhibit incurable ERS	Chen et al. (2023)
Rat, HK2	renal IRI	Baicalin	Inhibit incurable ERS	Lin et al. (2014a), Lin et al. (2014b)
Rat	renal IRI	Luteolin	Inhibit incurable ERS	Hong et al. (2017)

In controllable ERS, the UPR-related protection pathway predominates. According to research, the compound isoliquiritigenin, known for its antioxidant properties, triggers ERS and promotes hormesis as a defense mechanism against cisplatin-induced nephrotoxicity in LLC-PK1 pig kidney cells (Gómez-Sierra et al., 2020). When ERS is incurable and ER homeostasis is not regained, the UPR triggers the pathway leading to cell death. Inhibition of ERS may be a feasible option in most cases. TUDCA, a natural derivative of bile acid, has demonstrated the ability to inhibit ERS (Peng et al., 2015). TUDCA can reduce cell apoptosis and have a protective effect on renal IRI in both *in vivo* and *in vitro* conditions by blocking the expression of GRP78 and CHOP, which inhibits ERS (Gao et al., 2012). Treatment with naringenin activates the NRF2/HO-1 signaling pathway, preventing ERS and reducing pyroptosis and apoptosis, ultimately providing protective effects against renal IRI (Zhang B. et al., 2022). The ROS scavenger seems to be another option for ERS inhibition and AKI therapy. Regulation of NRF2/HO-1 signaling by the XBP1 pathway plays a role in the interaction between ERS and impaired mitochondrial function (Wu et al., 2014). Renal IRI could be ameliorated by the upregulation of the antioxidant response through NRF2/HO-1, which is achieved by reducing XBP1 levels in renal epithelial cells (Zhang et al., 2021). Suppression of ERS protein expression and mitigation of renal apoptosis occur by inhibiting FoxO4-induced ROS production. Inhibiting the ERS pathway has a protective impact on AKI by restraining ROS activity (Liu et al., 2019).

Chinese herbal medicines also alleviate AKI through ERS signaling. Curcumin decrease renal ERS and apoptotic protein biomarkers and mitigate nephrotoxicity in rats treated with gentamicin (Laorodphun et al., 2022). The binding ability of Forsythiaside A (FTA, acquired from *Forsythia Fructus*) to GRP78 has been demonstrated to be effective. In the mouse model of sepsis-induced AKI, FTA, as a potential suppressor of ERS, hinders the PERK pathway and enhances sepsis-induced AKI by exerting anti-inflammatory and anti-apoptotic impacts (Chen et al., 2023). Baicalin, a flavonoid glycoside derived from a traditional Chinese medicine, has demonstrated its ability to safeguard the rat kidney against IRI through the inhibition of apoptosis and inflammation (Lin et al., 2014a). The protective effect of Baicalin on H_2_O_2_-stimulated human renal tubular cell line HK2 is attributed to the suppression of ERS and the stimulation of NRF2 signaling (Lin et al., 2014b). Vegetables, fruits, and plants contain luteolin, which is a type of flavonoid. It has multiple biological properties and is protective in kidney disease (Domitrović et al., 2013; Arslan et al., 2016; Xin et al., 2016). The ERS inhibition effect of luteolin may be importance in improving renal IRI (Hong et al., 2017).

## 6 Conclusion

ERS is intricately involved in developing various acute conditions that contribute to AKI. ERS plays a critical role in human AKI cases and animal models of IRI injury. The interplay between ERS and AKI is multifaceted. ERS functions as a double-edged sword within the context of AKI. At moderate or chronic levels, it can trigger pro-survival cellular signaling pathways. But ERS can drive cell death when it reaches severe or acute levels. Of course, the mechanisms by which the ER plays a role in AKI need to be further studied. How important is endoplasmic reticulum stress in AKI? Is the treatment of AKI by regulating ER stress a direct or indirect effect? Is there a key target protein in ERS that can be evaluated for disease prognosis? The solution of these problems will be conducive to the development of ERS-related treatment measures. In summary, this review is important in unraveling the connection between ERS and AKI, thereby potentially uncovering new therapeutic approaches for the management of AKI.
